# Apoptosis of neutrophils, expression of TREM-1 on neutrophils and IL-17 responses in experimental burn in injury are related to the type and time of burn exposure

**DOI:** 10.1186/cc10617

**Published:** 2012-03-20

**Authors:** A Alexis, D Carrer, A Pistiki, K Louis, D Droggiti, J Van der Meer, M Netea, E Giamarellos-Bourboulis

**Affiliations:** 1 University of Athens, Medical School, Athens, Greece; 2 UMC St Radboud, Nijmegen, the Netherlands

## Introduction

To define inflammatory responses in experimental burn injury in relation with the type and time of burn exposure.

## Methods

Burn injury was induced in 110 C57/B6 male mice after time exposure of their back as follows: group 0, sham; group A, 60°C for 60 seconds; group B, 60°C for 45 seconds and 4°C for 45 seconds; group C, 75°C for 60 seconds; group D, 90°C for 5 seconds; and group E, 4°C for 45 seconds and 60°C for 45 seconds. Mice were sacrificed at 24 and 48 hours. Tissues were cultured and splenocytes were isolated and stimulated with heat-killed *Staphylococcus aureus *and *Candida albicans *for 5 days for release of IL-17. Neutrophil apoptosis and expression of TREM-1 were determined after staining for ANNEXIN-V, PI and anti-TREM-1-PE and flow cytometry analysis.

## Results

**Figure 1:**
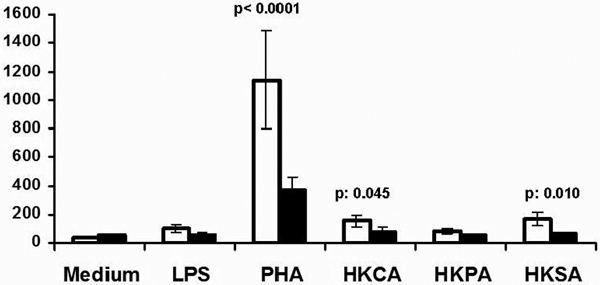
**Release of IL-17 by mice splenocytes in relation to the type of thermal injury**.

Mean respective apoptosis of groups 0, A, B, C, D and E at 24 hours were 37.9%, 77.6%, 81.9%, 73.8%, 83.6% and 75.4%; and at 48 hours 78.5%, 79.4%, 77.7%, 78.2%, 81% and 84.9% (*P *< 0.05 group 0 vs. others). Mean respective MFI of TREM-1 of groups 0, A, B, C, D and E at 24 hours were 2.4, 4.4, 3.4, 3, 3.2 and 3; and at 48 hours 2.7, 2.8, 2.8, 2.6, 2.8 and 2.7 (*P *< 0.05 group 0 vs. others). Tissue cultures were sterile. Release of IL-17 was greater by splenocytes of group D (Figure 1).

## Conclusion

Increased neutrophil apoptosis and TREM-1 expression and modulated IL-17 responses are found within burn injury.

